# Hyperbaric oxygen therapy for digital ulcers due to Raynaud’s disease

**DOI:** 10.1080/23320885.2018.1525684

**Published:** 2018-10-25

**Authors:** Tomoya Sato, Kiyohito Arai, Shigeru Ichioka

**Affiliations:** a Department of Plastic and Reconstructive Surgery, Saitama Medical University, Moroyama, Japan;; b Department of Cardiology, Saiseikai Kurihasi Hospital, Kuki City, Japan

**Keywords:** Addiction, alcohol, media, qualitative interviews, trust, knowledge

## Abstract

We present a case of Raynaud’s disease with digital ulcers that was successfully treated with hyperbaric oxygen therapy. Hyperbaric oxygen therapy can be considered as a safe and useful adjunct treatment for intractable digital ulcers in patients with Raynaud’s disease.

## Introduction

Raynaud’s phenomenon often becomes severe, resulting in digital ulcers, which are extremely painful and decrease the patient’s quality of life. The treatment for this disease is challenging because digital ulcers are often difficult to heal with conventional therapy. Herein, we present a case of Raynaud’s disease with digital ulcers that was successfully treated with hyperbaric oxygen therapy.

## Case Presentation

**Figure 1.**
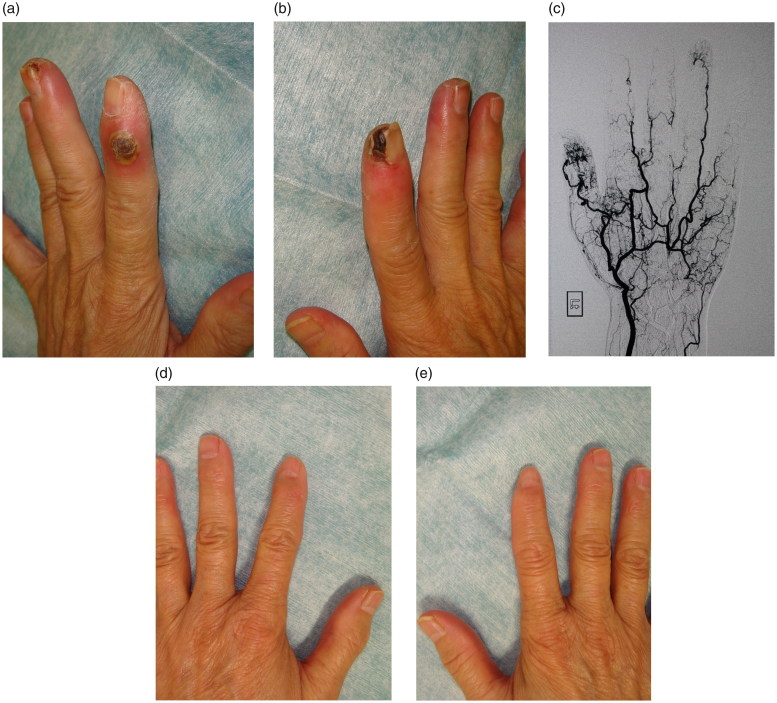
(a,b) Bilateral index fingers developed painful ulcers with eschar. (c) Angiogram shows poor distal runoff in digital arteries. (d,e) Twelve-month follow-up post hyperbaric oxygen therapy.

An 84-year-old woman presented with painful ulcers on her bilateral index fingers visited our hospital. She had been treated for interstitial pneumonia and Raynaud’s disease by a rheumatologist. She had a 5-year history of Raynaud’s phenomenon. Two months prior to the visit, she began to demonstrate peripheral cyanosis on her fingers and developed ulcerations on bilateral index fingers (Figure 1(a, b)). The ulcers were severely painful and were covered with black eschar, and her fingers were cold. Angiography findings revealed poor arterial perfusion in her fingers (Figure 1(c)). Blood test results indicated slightly increased inflammatory indexes, including an erythrocyte sedimentation rate of 15 mm/h and C-reactive protein of 0.34 mg/dL. Tests for antinuclear antibody, anti-dsDNA, anti-Sm, anti-SM/RNP, anti-Scl-70, anti-Jo-1, rheumatoid factor, anticentromere antibody, cytoplasmic antineutrophil cytoplasmic antibody, and myeloperoxidase antineutrophil cytoplasmic antibody revealed normal ranges. Skin biopsy did not show any specific findings.

In the outpatient clinic, she underwent conservative therapy with a calcium-channel blocker and anti-platelet drugs: oral administrations of nifedipine (20 mg/day), cilostazol (100 mg/day), and Beraprost (60 µg/day); however, the peripheral cyanosis and digital ulcers exacerbated. Thus, she was indicated hyperbaric oxygen therapy to improve digital ischemia.

The patient was admitted to our hospital and underwent hyperbaric oxygen therapy. The treatment protocol consisted of 100% oxygen at 2.0 atm of absolute pressure for 60 min. She underwent a total of 10 sessions of this therapy during 2 weeks of hospitalization. The patient had no side effects associated with the hyperbaric oxygen therapy. The cyanosis around the ulcers disappeared after the treatment. The pain due to the ulcers was remarkably reduced, and the patient required no painkillers at discharge. The ulcer size gradually decreased, and complete healing was accomplished 6 weeks after discharge. The patient experienced no recurrence in the 12-month follow-up period (Figure 1(d, e)).

## Discussion

Raynaud’s phenomenon is characterized by spasms of small arteries of the fingers and toes, which causes the skin to turn pale and subsequently dusky blue, often leading to skin ulcers or necrosis. Raynaud’s disease is categorized into two subtypes: primary Raynaud’s disease with unknown cause and secondary Raynaud’s disease with underlying neurovascular, hematological, drug-induced, or connective tissue disorders (scleroderma being the leading cause) [1]. Digital ulcers induced by severe Raynaud’s phenomenon are often refractory. Calcium channel blockers, vasodilators, and prostanoids are currently used for treating Raynaud’s phenomenon; however, such treatment is only effective in 16% of patients [2]. Digital sympathectomy is considered in patients with severe disease, but it is an invasive treatment.

Hyperbaric oxygen is a less invasive therapy that is used for elevating the arterial dissolved oxygen concentration and relieving the hypoxia of skin tissue by placing the patient under an oxygen pressure higher than the atmospheric pressure [3]. The resultant elevated arterial dissolved oxygen levels accelerate neovascularization by stimulating new blood vessel growth by local endothelial cells. It also enhances the recruitment and differentiation of circulating stem/progenitor cells to form blood vessels de novo. Levels of wound growth factors such as vascular endothelial growth factor, basic fibroblast growth factor, and angiopintin are also elevated, whereas monocyte chemokine synthesis is suppressed, and chronic inflammatory responses are diminished [4,5]. Certain guidelines and previous reports show effectiveness for chronic wound healing, especially in diabetic ulcers and ischemic ulcers, [6,7] whereas there is a paucity of data about chronic wounds due to vasculitis and collagen diseases [8]. Tissue ischemia is the primary cause of ulceration induced by Raynaud’s phenomenon; thus, tissue hypoxia can be relieved by supplying oxygen.

In conclusion, hyperbaric oxygen therapy can be considered as a safe and useful adjunct treatment for intractable digital ulcers in patients with Raynaud’s disease. Clinical studies are desirable for the evaluation of the efficacy on digital ulcers.
